# Antimicrobial Peptides as Potential Alternatives to Antibiotics in Food Animal Industry

**DOI:** 10.3390/ijms17050603

**Published:** 2016-05-03

**Authors:** Shuai Wang, Xiangfang Zeng, Qing Yang, Shiyan Qiao

**Affiliations:** State Key Laboratory of Animal Nutrition, Ministry of Agriculture Feed Industry Centre, China Agricultural University, Beijing 100193, China; wangshuai0919@cau.edu.cn (S.W.); zengxf@cau.edu.cn (X.Z.); yangqingcau@163.com (Q.Y.)

**Keywords:** antimicrobial peptides, antibiotic resistance, applications, swine, poultry

## Abstract

Over the last decade, the rapid emergence of multidrug-resistant pathogens has become a global concern, which has prompted the search for alternative antibacterial agents for use in food animals. Antimicrobial peptides (AMPs), produced by bacteria, insects, amphibians and mammals, as well as by chemical synthesis, are possible candidates for the design of new antimicrobial agents because of their natural antimicrobial properties and a low propensity for development of resistance by microorganisms. This manuscript reviews the current knowledge of the basic biology of AMPs and their applications in non-ruminant nutrition. Antimicrobial peptides not only have broad-spectrum activity against bacteria, fungi, and viruses but also have the ability to bypass the common resistance mechanisms that are placing standard antibiotics in jeopardy. In addition, AMPs have beneficial effects on growth performance, nutrient digestibility, intestinal morphology and gut microbiota in pigs and broilers. Therefore, AMPs have good potential as suitable alternatives to conventional antibiotics used in swine and poultry industries.

## 1. Introduction

Antibiotics are widely used for disease prevention and growth promotion in conventional livestock and poultry production [1]. In 2010, the global antibiotic consumption in food animal production was conservatively estimated at 63,151 tons [2]. Concurrent with the success of antibiotics for treating infections, the emergence and rapid dissemination of antibiotic-resistant bacteria poses substantial risks for human health. Antibiotic resistance has become an increasingly serious problem with global human deaths due to antibiotic resistant infections predicted to reach 10 million by 2050, more than the current death toll associated with different forms of cancer [3]. In addition, the number of approvals of new antibiotics has significantly and steadily decreased in the past three decades [4]. For this reason, there is an urgent need to develop novel antimicrobial agents, including alterative drugs based on antimicrobial peptides [5].

Antimicrobial peptides (AMPs) have been described as evolutionarily ancient weapons against microbial infections [6]. Produced by all organisms, from prokaryotes to human beings, AMPs serve a fundamental role in innate immunity. As an important component of the innate immune system, AMPs provide immediately effective, non-specific defenses against infections. Antimicrobial peptides are attractive candidates for the design of new antimicrobial agents for specific application because of their natural antimicrobial properties and a low propensity for the development of bacterial resistance [7]. In this article, we discuss the potential of AMPs as alternatives to conventional antibiotics, including their broad-spectrum of activity, low level of induced resistance, and immunomodulatory properties. The potential applications of AMPs in swine and broiler production are also reviewed.

## 2. Antimicrobial Peptides

Antimicrobial peptides are small biological molecules (<10 kDa) with a broad-spectrum of activity against bacteria, fungi, protozoa, and some viruses [8]. Unlike conventional antibiotics, which usually function through a defined high-affinity antimicrobial target and which can induce resistance in microorganisms, AMPs exert multiple antimicrobial activities that might provide a strategy to prevent bacteria from developing resistance [9]. Apart from directly attacking microbes, AMPs can confer protection by alternative mechanisms, such as maintenance of normal gut homeostasis, and modulation of host inflammatory responses [8,10]. The properties of AMPs include responding to microbial infections by acting on host targets rather than microbial targets which would be an advantage over the traditional antibiotics.

## 3. Structure of Antimicrobial Peptides

Antimicrobial peptides have certain common features. They are small molecules (12–50 amino acids) [8] containing positive charge and an amphipathic structure. Based on their secondary structure, AMPs fit into four major classes–namely, α-helical peptides, β-sheet peptides, extended peptides, and loop peptides [6]. The α-helical AMPs, including cecropins, magainins, and LL-37, are characterized by their structure, an extremely high propensity for the formation of α-helix. β-sheet AMPs are stabilized by two to four disulfide bridges and form relatively rigid structures, such as human α- and β-defensins, plectasin, and protegrins. The extended AMPs, which are rich in proline, tryptophan, arginine or histidine, do not fold into regular secondary structure (for example, indolicidin). The loop AMPs, including bactenecin, form loop structures owing to a disulfide bridge. Knowledge on the structures of AMPs will greatly help in the understanding of the mechanisms of action employed by AMPs and accelerate the generation of novel AMPs more suitable for pharmaceutical applications.

## 4. Broad-Spectrum Activity

Natural AMPs typically have a broad-spectrum activity against Gram-negative and Gram-positive bacteria, fungi, eukaryotic parasites and viruses [11]. In particular, a major strength of AMPs is their ability to kill multidrug-resistant bacteria. Zhang *et al.* [12] demonstrated that AMPs could inhibit methicillin-resistant *Staphylococcus aureus* and multidrug resistant *Pseudomonas aeruginosa*. In our laboratory, we also found that the antimicrobial peptide sublancin (artificial) has powerful inhibitory efficacy against methicillin-resistant *Staphylococcus aureus* (Unpublished data). The broad-spectrum activity of AMPs is one of their characteristics making them appropriate candidates for antibiotic alternatives.

### 4.1. Antibacterial Activity

Currently, the best-studied class of AMPs are those with antibacterial activity [13]. Most active AMPs are able to interact with bacterial membranes [14]. It is generally hypothesized that three main mechanisms could account for peptide permeation of the membrane of the target cell, including “barrel-stave model”, “carpet model” and “toroidal-pore model” [15,16]. In the “barrel-stave model”, the attached peptides aggregate to form a bundle with a central lumen and insert into the hydrophobic core of the membrane forming a trans-membrane pore [16]. The “carpet model” suggests that AMPs bind onto the phospholipid head covering the surface of membranes in a carpet-like manner and disrupt the bilayer curvature like a detergent beyond a threshold concentration of membrane-bound peptide [15]. The “toroidal-pore model” involves aggregation of peptide helices into the membrane, inducing the lipid monolayers to bend continuously through the pore so that both the inserted peptides and the lipid head groups line the water core [10]. Other mechanisms of antibacterial activity, including inhibition of cell-wall synthesis, suppression of protein and nucleic-acid synthesis, and inhibition of enzymatic activity, have been reviewed previously [14]. We will discuss the antibacterial activity of AMPs according to their origins.

Insect peptides are one of the largest groups of known AMPs. Cecropins constitute one of the most extensively studied AMPs among those synthesized by insects, and they are an important component of insect host defense systems against bacterial infection [17]. It has been reported that cecropins (synthetic) exhibited powerful inhibitory efficacy against *Escherichia coli*, *Pseudomonas aeruginosa*, *Bacillus megatherium*, and *Micrococcus luteus* [18]. The destruction of the integrity of the bacterial membrane has been suggested to be the mode of action for this class of peptides [19]. A second prominent group of insect AMPs is the insect defensins [20]. These peptides act against Gram-positive bacteria and participate in the antibacterial defense reactions in insects.

The amphibian skin contains a rich arsenal of broad-spectrum AMPs to defend against noxious microorganisms [21]. Magainins, isolated from the skin of the African clawed frog (*Xenopus laevis*), are a family of peptides with broad-spectrum antimicrobial activity. Magainin 2 (synthetic) displayed antibacterial activity against numerous Gram-negative and Gram-positive bacteria, such as *Escherichia coli*, *Staphylococcus aureus*, and *Klebsiella pneumonia* [6]. Limnochariin (synthetic), a novel antimicrobial peptide from the skins of amphibians, showed antimicrobial activities against 4 Gram-positive bacteria and 11 Gram-negative bacteria [22]. Hylaranins (synthetic), a new class of amphibian antimicrobial peptide from the skin secretions of the Oriental broad-folded frog, *Hylarana latouchii*, showed powerful antibacterial activity against *Escheriachia coli* and *Staphylococcus aureus* [23].

Defensins and cathelicidins are two principal classes of AMPs that have been identified in mammals. Defensins show a broad range of antimicrobial activity against bacteria that have demonstrated resistance to antibiotic treatment [24]. Human β-defensin 3 (HBD3) (artificial), isolated from the epidermal keratinocytes of psoriasis patients, possesses bactericidal activity against many potentially pathogenic microbes, such as multidrug-resistant *Staphylococcus aureus* and vancomycin-resistant *Enterococcus faecium* [25]. Cathelicidins exert antibacterial activity against both Gram-positive and Gram-negative bacteria via electrostatic interactions with the bacterial cell membrane [26,27]. The only cathelicidin peptide identified in humans is LL-37 (synthetic) and it has been shown to have considerable activity against *Pseudomonas aeruginosa*, *Salmonella typhimurium*, *Escherichia coli*, *Listeria monocytogenes*, and *Staphylococcus aureus* [28].

### 4.2. Antifungal Activity

Over 70 thousand taxonomically distinct fungi have been identified and some of these have potential to pose serious threats to human health [29]. *Candida albicans* is one of the major fungal pathogens that affects humans and can cause illnesses ranging from superficial mucosal infections to hematogenously disseminated candidiasis. Antimicrobial peptides have pleiotropic functions not only to display broad-spectrum antibacterial activity but also exert strong antifungal activity and could be useful in addressing fungal infections.

It was originally proposed that the mechanisms of action of AMPs against fungi involved fungal cell lysis and interference with fungal cell wall synthesis [30]. Cathelicidin peptides (synthetic), including SMAP-29, BMAP-27, BMAP-28, protegrin-1 and indolicidin, rapidly destroyed *Candida albicans* and *Cryptococcus neoformans* cells via membrane permeabilization and damage [31]. However, an increasing body of evidence suggests that AMPs show their effects through alternative modes of action. Vylkova *et al.* [32] found that human β-defensin 2 (hBD-2) (synthetic) and hBD-3 (synthetic) could destroy *Candida albicans* in an energy-dependent and salt-sensitive manner without causing gross membrane disturbance or lysis. However, the specific mechanisms of destruction remain to be elucidated. Research has shown that LL-37 could reduce the *C. albicans* attachment to abiotic surfaces, oral epidermis and murine urinary bladders by interacting with yeast carbohydrate and protein cell-wall components, which is of critical importance in prevention *C. albicans* colonization and infection by AMPs [33].

### 4.3. Antiviral Activity

Morbidity and mortality associated with viral infectious diseases is an escalating problem, especially with the emergence of drug-resistant viral strains. Thus, the development of novel and alternative antiviral agents is of great importance. Many AMPs have been reported as viral inhibitors [34]. The natural and safe antimicrobial peptide subtilosin (artificial) has shown potent virucidal and antiviral activity against Herpes simplex virus type 1 (HSV-1) [35]. The human cathelicidin, LL-37, and the murine cathelicidin, mCRAMP (synthetic), have been demonstrated to possess significant anti-viral activity against influenza virus both *in vitro* and *in vivo* [36]. The antiviral activity of AMPs appears to be related to direct interaction with the virion or is a result of an indirect effect through interactions with potential target cells. The antimicrobial peptide Dermaseptin S4 (synthetic) prevents human immunodeficiency virus (HIV) infection by disrupting the virion integrity [37]. Similarly, LL-37 can inhibit a variety of influenza A virus strains through a mechanism that mainly involves direct interactions with the virus [38]. Besides directly inactivating virus particles, AMPs block viral entry into cells by interaction with specific cellular receptors involved in virus internalization [39] or by antagonizing the virus proteins that fuse with target cells [40].

## 5. Low Level Induced Resistance to AMPs

Antimicrobial peptides have the ability to bypass the common resistance mechanisms that are reducing the usefulness and safety of conventional antibiotics. The novel antimicrobial peptide dendrimer G3KL (synthetic) was demonstrated to be a promising antimicrobial agent with antibacterial activity against multidrug-resistant and extensively drug-resistant *Acinetobacter baumannii* and *Pseudomonas aeruginosa* [41]. The antimicrobial peptide defensin (synthetic) from *Tribolium castaneum* displays *in vitro* and *in vivo* antimicrobial activity against drug resistant *Staphylococcus aureus* probably via disruption of the bacteria cell membrane [42]. In addition, the human antimicrobial peptide LL-37 exhibits significant antimicrobial activity against multidrug-resistant *Acinetobacter baumannii* [43].

However, it is inevitable that pathogens have evolved mechanisms that resist deleterious damage by AMPs. These include degradation of AMPs by production of proteolytic enzymes, repulsion of the peptides by alternation of net surface charges, expulsion of the peptides using membrane efflux pumps, and reducing the bacterial membrane fluidity through alternations in Lipid A. Even with such defensive mechanisms in pathogens, AMPs still provide protection. Compared with the conventional antibiotics, one of the strengths of AMPs is their low propensity for resistance development [44]. Three mechanisms are important in their low level of induced resistance. First, the positively charged peptides interact directly with the negatively charged cellular membranes of the target cells due to the electrostatic binding. The peptide-membrane interactions result in membrane permeabilization, which leads to a rapid cell death [45,46]. This physicochemical mechanism lessens development of bacterial resistance because the target of AMPs is the bacterial membrane and membrane redesign by bacteria would be a “costly” solution for most microbial species [6]; Secondly, AMPs have multiple mechanisms for attacking bacteria, increasing the probability of success and decreasing the probability of bacterial survival, which might be the best strategy to prevent bacteria from developing resistance [9]. It has been demonstrated that nisin (artificial) is a multi-function antimicrobial peptide with at least four different antimicrobial activities combined in one molecule, including inhibition of cell-wall synthesis, increasing pore formation in bacterial membranes, activation of cell wall autolytic enzymes and inhibition of bacterial spore germination [47]; Thirdly, increasing evidence suggests that AMPs are modulators of innate immunity. Because AMPs act through a diverse innate immune system rather than direct action on bacteria, increased resistance is less likely.

## 6. Immune Modulation

Increasing evidence suggests that AMPs protect hosts from bacteria by alternative mechanisms that are not related to their direct antimicrobial activity. It is well documented that AMPs are effector molecules of innate and adaptive immunity with modulation of pro- and anti-inflammatory responses, chemotactic activity, and direct effects on adaptive immunity [8,45,48]. Ren *et al.* [49] found that dietary supplementation with composite AMPs (artificial), which consist of swine defensin and a fly antimicrobial peptide, increased T cell populations, enhanced the proliferation function of T cells in the peripheral blood, and decreased percentages of apoptotic spleen cells. This study suggests that the cellular immune function was evidently improved in AMP-treated weaning piglets. Shan *et al.* [50] reported that lactoferrin (artificial) enhanced proliferation of peripheral blood and spleen lymphocytes and effectively increased serum IgG, IgA, IgM, and IL-2 in weaning piglets. In broilers, the pig antimicrobial peptides (artificial) and rabbit sacculus rotundus antimicrobial peptides (artificial) were shown to improve intestinal mucosal immunity [51,52]. Additionally, pig antimicrobial peptides were also shown to increase expression of secretory IgA in the intestinal tract of specific-pathogen-free chickens, strongly supporting the hypothesis that pig antimicrobial peptides can enhance the intestinal mucosal immunity [53].

## 7. Application in Non-Ruminant Nutrition

To date, the most prevalent use of AMPs has been in the preservation of foods [54]. However, with microbial resistance to conventional antibiotics occurring, AMPs have attracted increased attention from the swine and poultry industries. It was reported that a few AMPs, such as antimicrobial peptide-A3, P5 (synthetic), and cecropin AD (artificial) had beneficial effects on growth performance, nutrient digestibility, intestinal morphology as well as gut microbiota [55].

### 7.1. Improved Growth Performance

In recent years, AMPs have been being extensively evaluated as novel antimicrobial drugs in swine and poultry production. Previous studies have demonstrated the benefits of AMPs on the growth performance of swine and poultry (Table 1). For piglets, most studies with AMPs are focused on weaning pigs. An antimicrobial peptide lactoferrin isolated from milk has been shown to increase the average daily gain (ADG) and the efficiency of gain (G:F) of weaning piglets by 41.80% and 17.20%, respectively [56]. In addition, the fusion peptide lactoferricin-lactoferrampin (artificial) improved growth performance and decreased diarrhea incidence in weaning pigs. The effects of lactoferricin-lactofeffampin on the growth performance and the incidence of diarrhea were very similar with that of the antibiotic colistin sulfate [57]. It was also reported that the antimicrobial peptides A3 and P5 had beneficial effects on growth performance in weanling pigs and broilers [58,59,60,61]. Treatment with antimicrobial peptides A3 or P5 resulted in growth performance similar to antibiotic treatment. The administration of pig antimicrobial peptide in the drinking water or feed was also capable of promoting growth performance of broilers [52]. All these studies suggest that AMPs have potential as novel alternatives to antibiotics growth promoters.

### 7.2. Impact on Nutrient Digestibility and Gut Morphology

The growth promoting effect of AMPs has been shown to be related to improvement in nutrient digestibility. An antimicrobial peptide isolated from potato (artificial) has been demonstrated to linearly improve the total tract apparent digestibility (The apparent total tract digestibility for a given nutrient is calculated by subtracting the total tract outflow of that nutrient from the quantity ingested by the animal) of dry matter in weaning pigs [67]. Similarly, the antimicrobial peptides A3 and P5 were found to increase the apparent total tract digestibility of dry matter, crude protein and gross energy in weaning piglets and broilers [58,59,60,61]. Wen and He [66] reported that dietary supplementation with an antimicrobial peptide, a cecropin hybrid (artificial), increased the apparent digestibility of crude fat, increased nitrogen retention and improved apparent metabolizable energy in broilers. However, in studies on amino acid digestibility, AMPs had no effect on coefficient of apparent digestibility of essential and non-essential amino acids in weanling pigs [58,67].

Where improvements in growth performance of pigs or broilers have been noted, the support of AMPs for normal intestinal morphology may also be causative in the improvement in growth performance. Wu *et al.* [65] reported that a higher villus height to crypt depth ratio in the jejunum and ileum as well as higher villus height were observed in piglets fed the antimicrobial peptide cecropin AD compared with control piglets. Similar results were also observed in pigs treated by lactoferrampin-lactoferricin [57] or AMP-A3 [58]. In broilers, the beneficial effects of AMPs on gut morphology were also demonstrated. Wang *et al.* [68] found that the antimicrobial peptide sublancin increased the villus height in the duodenum and the villus height to crypt ratio in the jejunum despite the *Clostridium perfringens* challenge (Table 2). In addition, the antimicrobial peptide cecropin A-D-Asn (artificial) was also found to improve intestinal villus structures, which might be explained on the basis of its antimicrobial activity. However, the effects of antimicrobial peptide cecropin A-D-Asn on promotion of villus growth need to be evaluated in germ-free animals.

### 7.3. Modulation of Gut Microbiota

It is well documented that AMPs exert anti-bacterial, antifungal and antiviral activity in *in vitro* studies. Previous studies suggest that AMPs beneficially affect the host animal by improving its intestinal health and creating the microbial ecology that suppresses harmful microorganisms like *Clostridium* and by favoring proliferation of beneficial microorganisms, e.g., *Lactobacillus* and *Bifidobacterium* [57,58] (Table 3). In pigs, the antimicrobial peptide lactoferrin significantly reduced the total viable counts of *Escherichia coli* and *Salmonella*, and increased the *Lactobacillus* and *Bifidobacterium* counts in the small intestine compared with the control group [62]. The antimicrobial peptide cecropin AD was also shown to increase *Lactobacilli* counts in *E. coli* challenged piglets [65]. The beneficial effects of AMPs on gut microbiota have also been observed in broilers. Wang *et al.* [68] reported that the antimicrobial peptide sublancin significantly decreased the cecal *Clostridium perfringens* in challenged broilers. In addition, the *Lactobacilli* counts had a tendency to decrease with lincomycin treatment but were less affected by sublancin, which may be an advantage for AMPs compared to traditional antibiotics in regulation of gut microflora. However, extensive research will be required to compare the effects of AMPs and the currently used antibiotics on intestinal microbiota.

## 8. Conclusions

Properties of AMPs, such as broad-spectrum (antibacterial, antifungal, and antiviral) effects and low levels of induced resistance make AMPs promising alternatives to conventional antibiotics. These peptides have a strong potential for application as feed additives in swine and poultry production. Antimicrobial peptides have been demonstrated to improve growth performance, promote nutrient digestibility and gut health, positively alter intestinal microbiota, and enhance immune function in pigs and broilers. The beneficial effects of AMPs on growth performance are mostly due to their antimicrobial and immunomodulating activity, thereby promoting nutrient digestibility and health. With the development of technology and evolving knowledge, effective use of AMPs has good potential to improve efficiency of pig and broiler production. However, the fate of AMPs *in vivo* is still poorly understood. Therefore, there is a need for research evaluating the pharmacokinetics of AMPs.

## Figures and Tables

**Table 1:** Effects of antimicrobial peptides on the growth performance of swine and poultry.

Antimicrobial Peptide	Dose, mg/kg	Treatment Effects (%, Compared to Control)				References
		Animal	ADG ^a^	ADFI ^a^	G:F ^a^	
Antimicrobial peptide-A3	60	Weanling pigs	2	1	0	[58]
	90		5	2	5	
Antimicrobial peptide-P5	40	Weanling pigs	4	1	2	[59]
	60		8	3	5	
Lactoferrin	1000	Weanling pigs	34	17	15	[62]
	1000		42	21	17	[56]
Bovine lactoferrin	1250	Weanling pigs	16	15	0	[63]
	2500		13	13	0	
Bovine lactoferrin-lactoferrampin	100	Weanling pigs	24	17	6	[57]
Composite antimicrobial peptides	4000	Weanling pigs	−6	−17	15	[64]
Cecropin AD	400	Weanling pigs	4	1	3	[65]
Antimicrobial peptide-A3	60	Broilers	1	0	1	[61]
	90		4	2	2	
Antimicrobial peptide-P5	40	Broilers	4	2	2	[60]
	60		7	3	4	
Pig antimicrobial peptide	150	Broilers	19	2	17	[52]
	200		20	1	18	
Cecropin A-D-Asn	2	Broilers	0	−5	5	[66]
	4		2	−6	9	
	6		1	−16	20	
	8		-2	−14	14	

^a^ ADG, ADFI, and G:F are average daily gain, average daily feed intake, and gain:feed, respectively.

**Table 2:** The effect of sublancin on small intestinal morphology of broilers challenged with *Clostridium perfringens* (day 28) ^1,2^.

Item	Uninfected Control	Infected Control	Sublancin (mg/L of Water)			Lincomycin (mg/L of Water)	SEM	*p-*Value
			2.88	5.76	11.52	75		
Duodenum								
Villus height, μm	910.4 ^b^	880.2 ^b^	906.6 ^b^	1016.3 ^a,b^	1104.0 ^a^	1144.0 ^a^	34.61	<0.01
Crypt depth, μm	188.7	197.1	177.8	192.4	186.8	179.2	6.72	0.35
Villus height:crypt depth	4.85 ^b,c^	4.46 ^c^	5.14 ^b,c^	5.29 ^b,c^	5.92 ^a,b^	6.44 ^a^	0.24	<0.01
Jejunum								
Villus height, μm	805.2	776.4	873.6	903.2	918.8	927.5	35.96	0.07
Crypt depth, μm	159.1	180.1	146.5	168.7	174.8	158.4	8.62	0.14
Villus height:crypt depth	5.14 ^a,b^	4.32 ^b^	6.11 ^a^	5.45 ^a,b^	5.26 ^a,b^	5.88 ^a^	0.35	0.03
Ileum								
Villus height, μm	588.0	576.0	608.7	624.1	651.4	544.6	36.16	0.41
Crypt depth, μm	134.9 ^a,b^	146.7 ^a^	130.4 ^a,b^	136.1 ^a,b^	123.6 ^a,b^	100.4 ^b^	8.64	0.02
Villus height:crypt depth	4.40	3.97	4.79	4.74	5.30	5.55	0.40	0.14

^1^ Uninfected control: Without *Clostridium perfringens* challenge. Infected control: *C. perfringens* challenge but no drug treatment. Sublancin (mg/L of water): *C. perfringens* challenge groups supplemented with sublancin at 2.88, 5.76, or 11.52 mg activity/L of water. Lincomycin (mg/L of water): *C. perfringens* challenge group supplemented with lincomycin at 75 mg/L of water; ^2^ *n* = 6; ^a–c^ Values in the same row with different superscripts differ (*p* < 0.05).

**Table 3:** Effects of antimicrobial peptides on the gut microbiota in swine and poultry.

Antimicrobial Peptide	Animal	Treatment Effects	References
Lactoferrin	Weanling pigs	Reduced total viable counts of *E. coli* and *Salmonella* in the small intestine	[62]
Bovine lactoferrin-lactoferrampin	Weanling pigs	Decreased the counts of *E. coli* in the ileum, caecum and colon and increased the counts of *Lactobacilli* and *Bifidobacteria* in the ileum, caecum and colon	[57]
Antimicrobial peptide-A3	Weanling pigs	Reduced total anaerobic bacteria, coliforms and *Clostridium* spp. in the ileum, cecum and feces	[58]
Antimicrobial peptide-P5	Weanling pigs	Reduced fecal and intestinal coliforms and caecal *Clostridium* spp.	[59]
Potato protein	Weanling pigs	Decreased viable counts of total bacteria, coliforms and *Staphylococcus* spp. in caecum and rectum	[67]
Cecropin AD	Weanling pigs	Decreased total aerobes while increasing total anaerobes in the ileum and increased the numbers of Lactobacillus in the cecum	[65]
Recombinant plectasin	Weanling pigs	Increased the abundance of Bifidobacterium in the ileum	[69]
Cecropin A-D-Asn	Broilers	Decreased aerobic bacteria counts in both jejunal and caecal digesta	[66]
Antimicrobial peptide-A3	Broilers	Reduced coliforms and *Clostridium* spp. counts in feces	[61]
Antimicrobial peptide-P5	Broilers	Reduced excreta total anaerobic bacteria and coliforms	[60]
Sublancin	Broilers	Reduced *Clostridium perfringens* in the cecum	[68]
